# Acute Myeloid Leukemia with t(8;21)(q22;q22) and Trisomy 4: A Rare Occurrence in a Female Child

**DOI:** 10.7759/cureus.3885

**Published:** 2019-01-14

**Authors:** Shawana Kamran, Sara A Awan, Kamran N Ahmad, Yasir Iqbal

**Affiliations:** 1 Hematology, Shifa International Hospital, Islamabad, PAK; 2 Hematology, Pakistan Air Force Hospital, Islamabad, PAK; 3 Pediatric Oncology, Shifa International Hospital, Islamabad, PAK

**Keywords:** acute myeloid leukemia, trisomy 4, cytogenetics, oncology, pediatrics

## Abstract

Acute myeloid leukemia (AML) with balanced translocation t (8;21) is one of the most frequent chromosomal abnormalities and carries a favorable clinical outcome. However, according to a literature review, additional chromosomal aberrations can affect the overall disease prognosis. Trisomy 4 is a rare numerical abnormality in AML patients with t (8;21), which can be associated with c-KIT gene involvement. In adults, c-KIT mutation carries an unfavorable clinical outcome; however, its incidence and clinical importance in the pediatric population are still under scrutiny. Here, we report a case of AML with t(8;21) and trisomy 4 in an eight-year-old female child and the clinical course of the disease.

## Introduction

Acute leukemia is one of the most common childhood cancers, however, acute myeloid leukemia (AML) constitutes only 15% to 20% of such cases [1]. In Pakistan, multicenter studies are needed to demonstrate the overall incidence and survival of children with AML. t(8;21) is a frequently oc­curring aberration in acute AML. It involves the fusion of the RUNX1 (runt-related transcription factor 1) gene on chromosome 21q22 and the RUNX1T1 (runt-related transcription factor 1; translocated to 1) gene on chromosome 8q22, resulting in the formation of the hybrid gene RUNX1/RUNX1T1. AML with t(8;21)(q22;q22), RUNX1/RUNX1T1 generally shows maturation in the myeloid lineage and is found in approximately 5% of cases of AML [2]. It usually has a good prognostic impact but some cases can have additional chromosomal aberrations, changing the overall disease prognosis. These include loss of sex chromosomes in almost 50% cases. In other instances, del 7q, del 9q, or +8 can occur. However, trisomy 4 is a very rare occurrence. The prognostic significance of trisomy 4 in AML is still not well-established. t(8;21)(q22;q22) along with trisomy 4 can have mutations of the c-KIT gene located in region 4q11-q12. KIT receptor tyrosine kinase mutations in adults with core binding factor acute myeloid leukemia9 (CBF-AML) are implicated as a prognostic factor, however, in the pediatric population, its prevalence and prognostic impact is still a matter of debate.

## Case presentation

An eight-year-old female presented with a three-day history of epistaxis and fever. On examination, there was no hepatosplenomegaly or lymphadenopathy. She underwent a bone marrow biopsy at an outside institution, where she was diagnosed as acute promyelocytic leukemia (APML) on morphology. Subsequently, she received three doses of all-trans-retinoic acid (ATRA). Her bone marrow aspiration slides and trephine block was referred to our institution for a second opinion. On presentation to our institution, laboratory investigations, including a complete blood count and coagulation studies, were ordered. Her complete blood count showed leukocytosis, with the differential leukocytic count revealing 89% blasts. This was compounded with moderate anemia according to the World Health Organization (WHO) guidelines for her age [3] and thrombocytopenia. Her laboratory investigations on presentation are shown in Table 1.

**Table 1:** Laboratory investigations on presentation

Test	Result	Normal Reference Range
Hemoglobin	10.10 g/dL	11.5-14.5 g/dL
White blood cells count	162000/μL	4000-12000/μL
Neutrophils	5%	30-55%
Lymphocytes	4%	40-60%
Monocytes	2%	1-4%
Eosinophils	0%	1-2%
Basophils	0%	0-0.75%
Blasts	89%	0%
Platelets count	86000/μL	150000-400000/μL
Prothrombin time	10.90 seconds	9.5-11.7 seconds
Activated partial thromboplastin time	22.40 seconds	24.8-36.2 seconds
International normalized ratio	1.0	0.8-1.3
Fibrinogen level	215.8 mg/dL	199-409 mg/dL

**Figure 1:**
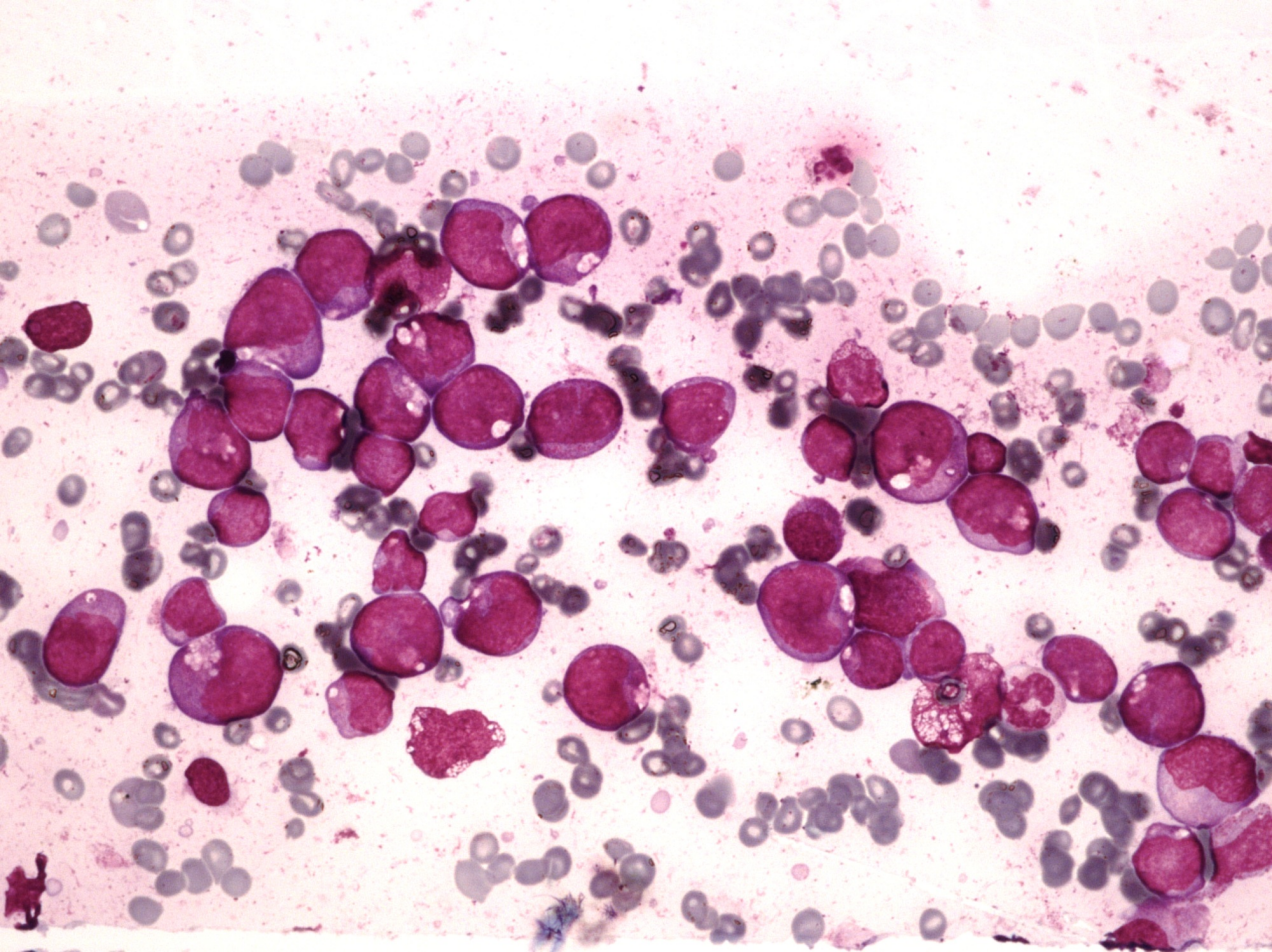
Bone marrow aspirate Bone marrow aspirate showing significantly increased blasts having immature chromatin, prominent nucleoli, and abundant cytoplasm. Cytoplasm has increased granules, Auer rods, and cytoplasmic vacuolation.

On review, her bone marrow aspirate revealed a hypercellular specimen with 76% blasts. These blasts were medium to large in size, with immature chromatin and abundant, hypergranular cytoplasm. They also showed cytoplasmic vacuolations, prominent nucleoli, and Auer rods. However, abnormal promyelocytes and Faggot cells (Figure 1) diagnostic of APML were not seen. The trephine biopsy showed hypercellular marrow with blasts comprising more than 90% marrow cellularity.

**Figure 2:**
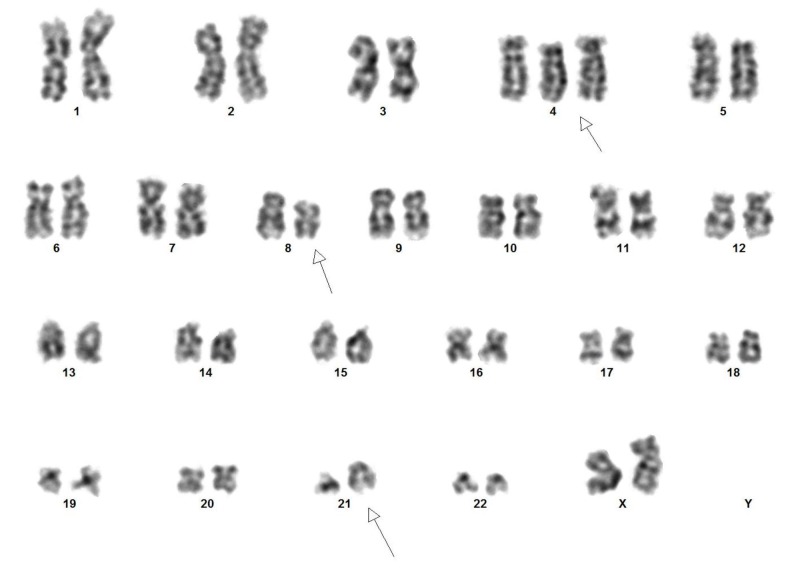
Karyotype analysis showed t(8;21)(q22;q22) with trisomy 4

A flow cytometric analysis was done on peripheral blood, which showed these blasts to be positive for CD13, CD33, and CD117, strongly positive for myeloperoxidase (MPO), had low expression of CD34, and were negative for HLA-DR. Chromosomal analysis was performed on unstimulated short-term peripheral blood cultures (24 hours), followed by Giemsa trypsin banding. Cytogenetic findings were described according to the International System for Human Cytogenetic Nomenclature (ISCN 2013). An analysis was performed on 16 metaphases, which showed 46,XX,t(8;21)(q22;q22)/47,idem,+4/46,XX. Figure 2 demonstrates the karyotype analysis of the patient.

Peripheral blood was used for RNA extraction using reverse transcription polymerase chain reaction (RT-PCR) protocols for PML-RARα and RUNX1/RUNX1T1 amplification. Polymerase chain reaction (PCR) for RUNX1/RUNX1T1 was positive and PML-RARα and core-binding factor subunit beta (CBFB) were negative. Deoxyribonucleic acid (DNA) analysis for c-KIT mutation was not performed due to the non-availability of the test.

Consequently, the patient was labeled as WHO category acute myeloid leukemia with t(8;21)(q22;q22);RUNX1/RUNX1T1 and was admitted to the oncology ward. She received conventional chemotherapy through the standard protocol. A repeat bone marrow biopsy was performed on day 29 post-chemo-induction, which revealed bone marrow in morphological and cytogenetic remission.

Following this, the patient was put on maintenance therapy. However, she presented 10 months later to our emergency department with disease relapse. Cytogenetics showed the persistence of the t(8;21) clone with no trisomy 4 seen. The patient was advised regular follow-up at the hematology clinic where workup for hematopoietic stem cell transplant was started.

## Discussion

The balanced translocation t(8;21) (q22;q22), RUNX1-RUNX1T1 is one of the most frequent chromosomal abnormalities in pediatric AML cases and confers a good prognosis. However, its occurrence with trisomy 4 is rare and studies elucidating the prognostic significance of this aberration are limited. In our study, the patient initially went into remission but the disease relapsed after 10 months. A similar case was reported in Malaysia, where an adult woman was suspected as having AML M3 according to the French-American-British (FAB) classification based on the initial presentation. However, her cytogenetic studies excluded the diagnosis of AML M3 and confirmed AML with trisomy 4 in addition to t(8;21). The patient received all-trans-retinoic acid but died soon afterward. This raises concerns over the clinical outcome of patients with the aforementioned chromosomal abnormalities [4].

Pan et al. performed a retrospective analysis of 21 cases of acute leukemia with trisomy 4 published in 2007. It showed that acute leukemia with trisomy 4 has distinctive clinical and laboratory features and a poor prognosis. The most common AML subtype with trisomy 4 was identified as M2 [5].

A study was conducted on 94 adults (age 15 or above) in Japan where patients with t(8;21) AML were further categorized according to additional chromosomal aberrations. Twenty-seven cases (29.3%) of t(8;21) AML with loss of the sex chromosome and 10 cases (10.6%) of t(8;21) AML with chromosome 9 abnormalities were identified. However, the study observed no significant difference in the clinical outcome of the aforementioned patients with those that had t(8;21) AML with no additional chromosomal aberrations. Trisomy 4 was found in only three cases (3.2%) and all these patients died within 2.4 years. These observations suggest that t(8;21) with trisomy 4 has a distinctive clinical outcome and may constitute a unique subtype of t(8;21) AML [6].

A German study reviewed the karyotypes of 916 pediatric patients with t(8;21) AML. The results concluded that trisomy 4 was found in 21 patients and these patients showed an inferior cumulative incidence of relapse and survival [7]. Chilton et al. also conducted a study on 87 patients with AML and trisomy 4 to determine the prognostic impact of this abnormality. Most adults (82%) and all pediatric patients (100%) with trisomy 4 achieved complete remission. Relapse occurred in 22% of trisomy 4 patients, however, they showed a similar relapse rate of 54% at five years to the comparator group (composed of AML patients with normal karyotype, classified as intermediate risk). The study observed that pediatric patients with trisomy 4 were more likely to relapse than their age-specific comparator group (60% vs 37%, P=0.06) and most relapsed within the first 12 months following diagnosis [8].

While the poor prognosis of patients of AML with t(8;21) and trisomy 4 is still being researched, some studies attribute this to the presence of the c-KIT gene located at chromosome 4. Trisomy 4 may lead to duplication and increased dosage of the mutated c-KIT allele, leading to a distinctive mechanism of leukemogenesis [9]. Beghini et al. reported a study where different KIT mutations were detected in four of the eight adult core binding factor acute myeloid leukemia (CBFL) patients and one childhood AML case bearing trisomy 4 with or without t(8;21). In three of the trisomy 4 cases, it was demonstrated that trisomy 4 leads to the duplication of the KIT mutated allele [10]. A Japanese study suggested an association between c-KIT mutations and trisomy 4 and further reported that KIT mutations are strongly associated with a poor prognosis in pediatric t(8;21) AML [11]. A study conducted in Greece also reaffirmed the association of KIT (mapped to 4q11) mutations with poor prognosis in pediatric AML with t(8;21) [12].

AML in the pediatric population shows a high degree of relapse, therefore, accurate prognostic markers are required to allow treatment modification. AML with different cytogenetic subgroups may respond differently to specific therapies. Therefore, performing cytogenetic studies in all patients with pediatric AML at diagnosis and during the course of the disease may lead to minimizing the risk of disease relapse.

## Conclusions

Chromosome abnormalities are important parameters for diagnosis, determining prognosis and treatment modalities in pediatric AML. Our findings illustrate the importance of cytogenetic studies in the diagnosis and risk stratification of acute myeloid leukemia in countries where comprehensive molecular testing is not available. In light of the literature review, AML with t(8;21) and trisomy 4 in pediatric patients should be closely monitored for risk of relapse.

## References

[1] Neglia JP (1988). Epidemiology of the childhood acute leukemias. Pediatr Clin North Am.

[2] Kroes W, Stevens-Kroef M (2017). t(8;21)(q22;q22) RUNX1/RUNX1T1. Atlas Genet Cytogenet Oncol Haematol.

[3] (2018). Haemoglobin concentrations for the diagnosis of anaemia and assessment of severity. https://www.who.int/vmnis/indicators/haemoglobin.pdf.

[4] Phan CL, Ong TC, Chang KM, Zubaidah Z, Puteri Jamilatul NMB (2010). Concomitant t(8;21) and trisomy 4 in a patient with acute myeloid leukemia (AML). Medicine & Health.

[5] Pan JL, Xue YQ, Qiu HY (2007). Clinical and experimental retrospective analysis on acute leukemia with trisomy 4 cell [Article in Chinese]. Zhonghua Yi Xue Yi Chuan Xue Za Zhi.

[6] Nishii K, Usui E, Katayama N (2003). Characteristics of t(8;21) acute myeloid leukemia (AML) with additional chromosomal abnormality: concomitant trisomy 4 may constitute a distinctive subtype of t(8;21) AML. Leukemia.

[7] Klein K, Kaspers G, Harrison CJ (2015). Clinical impact of additional cytogenetic aberrations, cKIT and RAS mutations, and treatment elements in pediatric t(8;21)-AML: results from an international retrospective study by the International Berlin-Frankfurt-Münster Study Group. J Clinical Oncol.

[8] Chilton L, Hills RK, Burnett AK, Harrison CJ (2016). The prognostic significance of trisomy 4 in acute myeloid leukaemia is dependent on age and additional abnormalities. Leukemia.

[9] Langabeer SE, Beghini A, Larizza L (2003). AML with t(8;21) and trisomy 4: possible involvement of c-kit?. Leukemia.

[10] Beghini A, Ripamonti CB, Cairoli R (2004). KIT activating mutations: incidence in adult and pediatric acute myeloid leukemia, and identification of an internal tandem duplication. Haematologica.

[11] Shimada A, Taki T, Tabuchi K (2006). KIT mutations, and not FLT3 internal tandem duplication, are strongly associated with a poor prognosis in pediatric acute myeloid leukemia with t(8;21): a study of the Japanese Childhood AML Cooperative Study Group. Blood.

[12] Manola KN (2009). Cytogenetics of pediatric acute myeloid leukemia. Eur J Haematol.

